# Complete cleft of the upper limb: A very rare anomaly

**DOI:** 10.4103/0970-0358.44842

**Published:** 2008

**Authors:** Yogesh C. Bhatt, Harpreet S. Bakshi, Kinnari A. Vyas, Girish S. Ambat, Hitesh Laad, Nikhil S. Panse

**Affiliations:** Department of Plastic Surgery, SSG Hospital and Medical College, Vadodara, Gujarat-390 001, India

**Keywords:** Complete splitting of upper limb, complete cleft upper limb, very rare congenital deformity

## Abstract

**Background:**

We describe here a very rare congenital deformity that involves the splitting of the right upper limb with the superior limb articulating with the shoulder joint, and the inferior limb laterally attached to the chest wall.

**Material and Methods:**

The child with this rare split limb was treated by transferring the inferior limb on an islanded pedicle to the superior one while creating the hand.

**Results:**

A unified limb was reconstructed while creating the hand without any compromise on the existing function and vascularity. The patient is on regular follow-up and further staged procedures have been planned to provide a better functional and aesthetic limb.

**Conclusion:**

The congenital deformity described here has not been mentioned in world literature so far and its embryological basis is a matter of discussion. Opinions regarding further management of this anomaly are invited from experts in the field.

## INTRODUCTION

We describe here a case of congenital complete cleft of the upper limb: a split upper limb in which correction was achieved by pedicled transfer of the inferior part of the limb. No case of this kind has been reported to date.

## CASE REPORT

**Figure 1:**
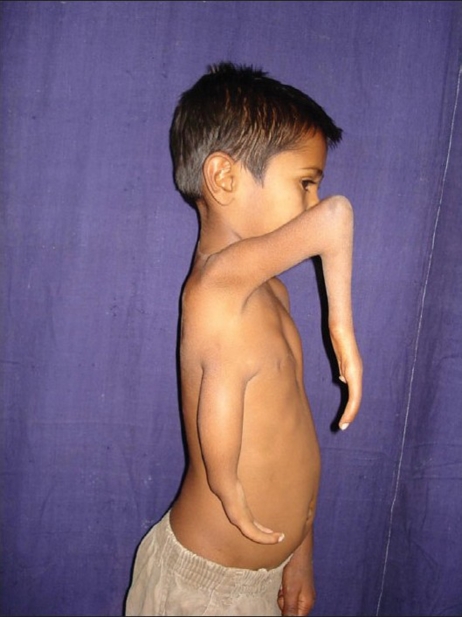
Pre-operative picture showing split Right upper limb

**Figure 2:**
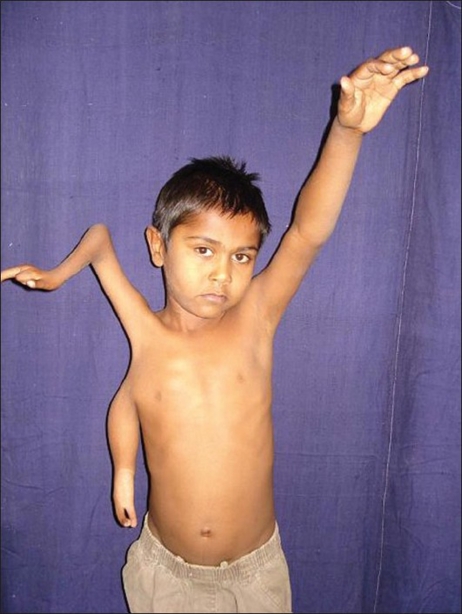
Pre-operative picture showing split Right upper limb

**Figure 3:**
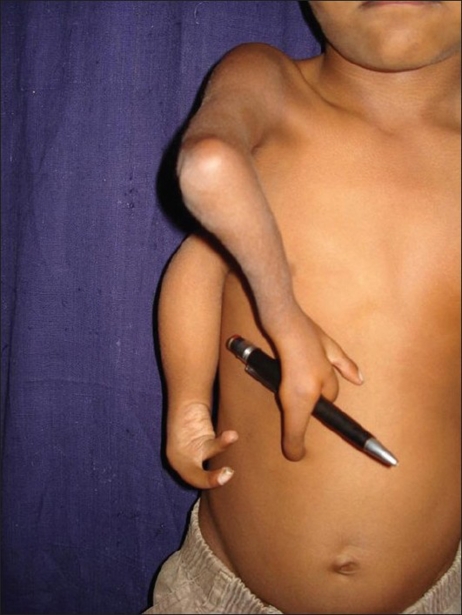
Pre-operative picture showing hand function

A six year-old boy presented with a congenital anomaly of two upper limbs on the right side [Figures 1–3]. Clinical examination revealed that the superior limb was articulating with the shoulder joint, and the inferior limb laterally attached to the chest wall. Both these limbs on the right side were hypoplastic compared to the left upper limb. The superior limb was approximately 7 cm shorter than the opposite limb (normal side), the superior limb being 33 cm in length from the shoulder joint while the inferior limb was 18 cm from the attachment with the chest wall. The skin over the forearm on the superior limb was shiny and atrophic with very little soft tissue mass compared with the inferior limb which was bulkier with normal-appearing skin. The superior limb was articulating with the torso via a normal shoulder joint. The scapula was small compared to that on the opposite side. The inferior limb was attached to the lateral thoracic wall by soft tissue at the level of the 4^th^ intercostal space. The superior limb had a humerus, a single bone at the forearm level, and a duplicated thumb with an index finger. There was synostosis at the elbow joint and the wrist joint was stable. All fingers showed symphalyngism; the middle ray including the 3^rd^ metacarpal was absent. The inferior limb had two fingers and a single bone with which it was attached to the chest wall. Clinically, the superior limb presented a picture of an ulnar hypoplastic hand where there was a stable wrist joint and a fused elbow joint. The inferior limb presented like a radial club hand with instability of the wrist joint. All movements were possible at the shoulder joint level but none were possible at the elbow joint and the wrist joint in the superior limb. Hand function was better in the inferior limb. The hand flexors were stronger in the inferior limb while the extensors were stronger in the superior limb. The clavicular head of the pectoralis was attached to the superior limb while the sternal head was attached to the inferior limb; the latissmus dorsi was inserted into the inferior limb.

### Investigations

**Figure 4:**
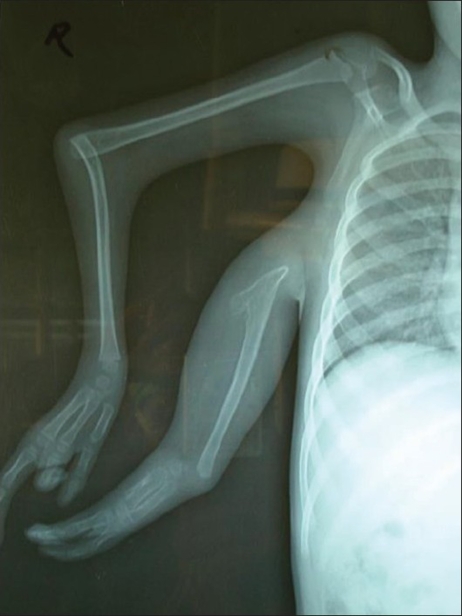
X-ray picture

An X-ray was taken of the right upper limb [Figure 4], and color Doppler, and MR Angiography were done.

The X-ray [Figure 4] revealed that the scapula on the right side was hypoplastic and the superior limb had a shoulder joint with elbow joint synostosis.

*Color Doppler* study [Figure 14] showed duplication of the right subclavian artery with separate origin of the inferior accessory subclavian artery. There was a patent radial artery with a inner diameter of 1.7 mm in the superior limb (radial) whereas there was a patent ulnar artery with a inner diameter of 2.0 mm in the inferior limb.

*Magnetic Resonance Angiography* [Figure 15] confirmed the findings of the color Doppler and the duplication of the subclavian vessels was found to arise from the infraclavicular level. The duplicated vessels running to the inferior limb were running along the lateral chest wall into the limb; the nerves also coursed along with the vessels.

### Planning and Operative procedure

Our goal was to transfer the inferior limb to the superior forearm as a neurovascular island, and to recreate the hand without compromising the existing function and vascularity.

### Operative procedure

An encircling incision was made around the inferior limb, the pectoralis major and the latissmus dorsi muscle attachments were divided, and the neurovascular pedicle was dissected and mobilised up to its anomalous origin in the infraclavicular region from the subclavian vessels. The inferior limb was then transferred to the superior forearm.

**Figure 5:**
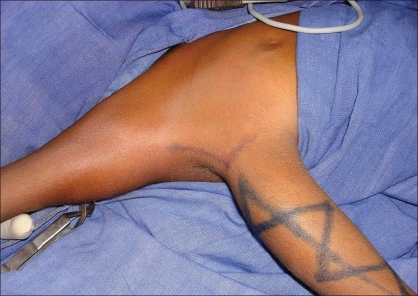
Zig-zag incision marking over the inferior limb

**Figure 6:**
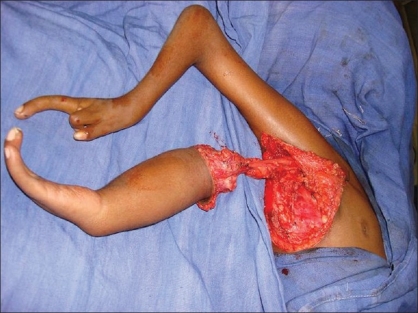
Encircling incision placed around the inferior limb

**Figure 7:**
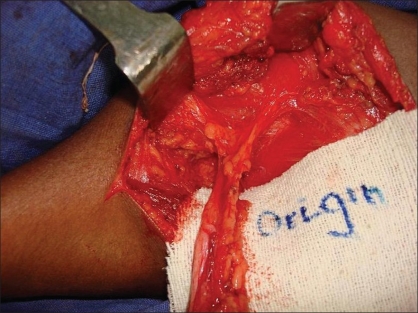
Dissection of pedicle upto its anomolous origin in infra-clavicular region from subclavian artery

**Figure 8:**
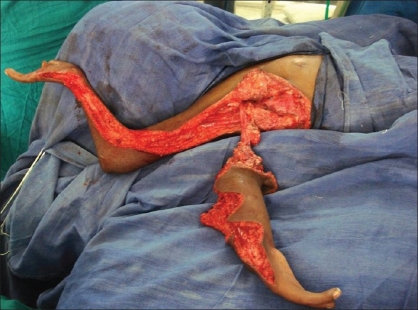
Zig-zag incisions on corresponding sides of both limbs

**Figure 9:**
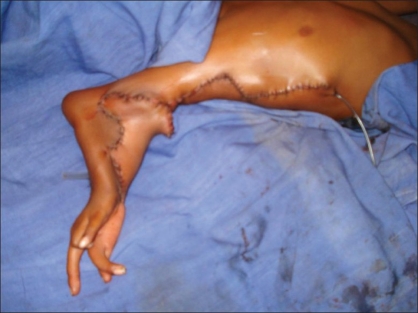
Transfer of inferior limb along the superior limb with creation of the hand

Zigzag incisions were made on corresponding sides of the skin on the forearm and hand. The transverse metacarpal ligament was created and carpal bones were approximated with nonabsorbable sutures. [Figures 5–9]

**Figure 10:**
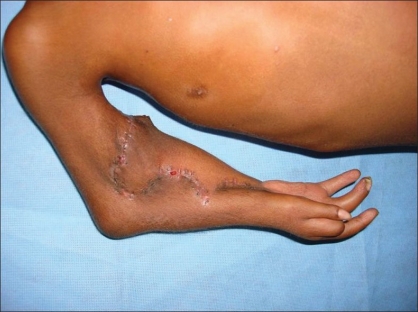
Post-operative picture

**Figure 11:**
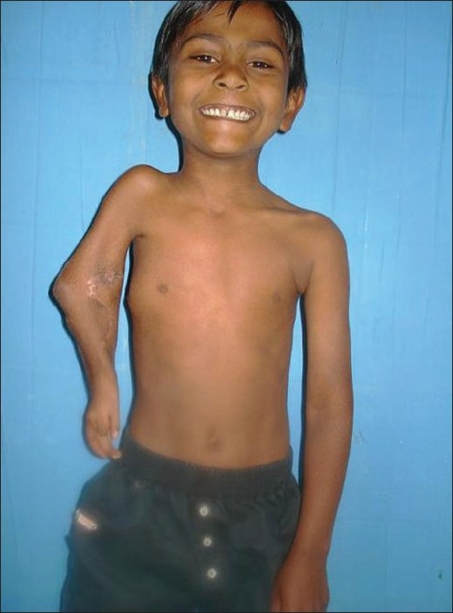
Post-operative picture

**Figure 12:**
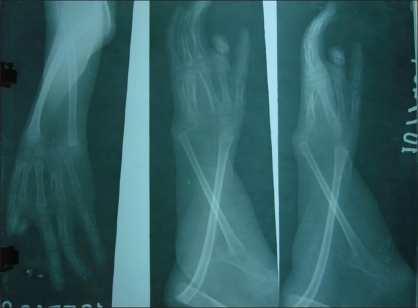
post-op. X-ray picture

The postoperative period was uneventful and the existing hand functions [Figures 10–13] were preserved. However, the abduction and external rotation at the shoulder joint was reduced because of the tautness of the transferred neurovascular pedicle and the bulk added at the forearm level.

**Figure 13A:**
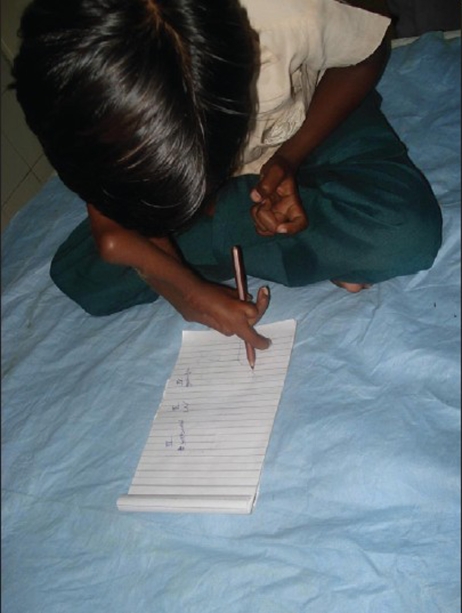
Post -op. photograph showing hand function

**Figure 13B:**
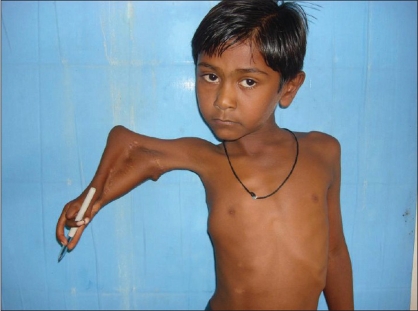
Post-operative photograph showing hand function

**Figure 14:**
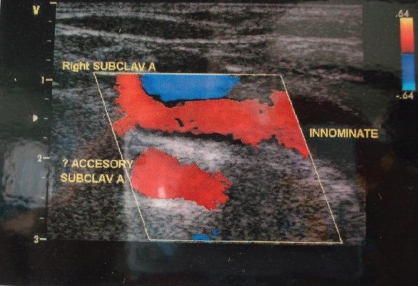
Color Doppler study showing duplication of right subclavian artery with separate origin of inferior accessory subclavian artery

**Figure 15:**
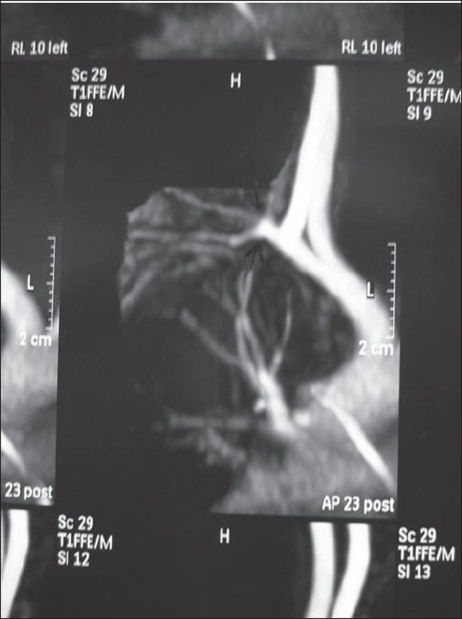
MR Angiography showing duplication of subclavian vessels Figure

Physiotherapy and re-education in terms of holding objects has been started early. The patient has been kept on regular follow-up and further staged procedures like distraction and synostosis of the elbow joint and debulking have been planned to provide a better functional and aesthetic limb. (**See video on**

.)

## DISCUSSION

The case described here has not been reported to date, hence, certain related cases deserve a mention.

*Cleft hand:* The true (typical) cleft hand[1,2] is characterized by a V-shaped cleft in the center of the hand associated with the absence of one or more digits. Syndactyly of the digits bordering the cleft, an abnormal first web space and transverse bones within the hand are seen. The condition may be unilateral or bilateral, and may involve the feet.[3]

*Mirror hand:* Mirror hand or ulnar dimelia is characterized by a limb with two ulnae, no radius, seven or eight fingers, and no thumb. The wrist and the elbow appear thick and the arm appears short.[4–8] The proximal elements of the forearm may be abnormal. The two ulnae are parallel, but rotated approximately 120 degrees to each other, and do not flex on the humerus. The wrist is almost always held in flexion and is frequently deviated to one side or the other.[9]

We have described a very rare congenital anomaly that involves complete splitting of the right upper limb with the superior limb articulating through a shoulder joint, while the inferior limb was attached to the chest wall laterally by soft tissue. The inferior limb was transferred to the superior one on an islanded pedicle while creating the hand.

The congenital anomaly described here has not been reported in the world literature to the best of our knowledge. Its embryological basis is a matter of discussion and opinion regarding its further management is invited from the experts in the field.
